# Antimicrobial Usage Factors and Resistance Profiles of Shiga Toxin-Producing *Escherichia coli* in Backyard Production Systems From Central Chile

**DOI:** 10.3389/fvets.2020.595149

**Published:** 2021-01-15

**Authors:** Erika Pavez-Muñoz, Camilo González, Bastián Fernández-Sanhueza, Fernando Sánchez, Beatriz Escobar, Romina Ramos, Verónica Fuenzalida, Nicolás Galarce, Gabriel Arriagada, Víctor Neira, Jeannette Muñoz-Aguayo, Cristian Flores-Figueroa, Timothy J. Johnson, Raúl Alegría-Morán

**Affiliations:** ^1^Departamento de Medicina Preventiva Animal, Facultad de Ciencias Veterinarias y Pecuarias, Universidad de Chile, Santiago, Chile; ^2^Instituto de Ciencias Agroalimentarias, Animales y Ambientales—ICA3, Universidad de O'Higgins, Rancagua, Chile; ^3^Mid-Central Research and Outreach Center, University of Minnesota, Saint Paul, MN, United States; ^4^Department of Veterinary and Biomedical Sciences, University of Minnesota, Saint Paul, MN, United States; ^5^Facultad de Ciencias Agropecuarias y Ambientales, Universidad Pedro de Valdivia, Santiago, Chile

**Keywords:** antimicrobial resistance, Shiga toxin-producing *Escherichia coli*, backyard production systems, zoonoses, one health, antimicrobial use

## Abstract

Shiga toxin-producing *Escherichia coli* (STEC) is a zoonotic pathogen and important cause of foodborne disease worldwide. Many animal species in backyard production systems (BPS) harbor STEC, systems characterized by low biosecurity and technification. No information is reported on STEC circulation, antimicrobial resistance (AMR) and potential drivers of antimicrobial usage in Chilean BPS, increasing the risk of maintenance and transmission of zoonotic pathogens and AMR generation. Thus, the aim of this study was to characterize phenotypic and genotypic AMR and to study the epidemiology of STEC isolated in BPS from Metropolitana region, Chile. A total of 85 BPS were sampled. Minimal inhibitory concentration and whole genome sequencing was assessed in 10 STEC strain isolated from BPS. All strains were cephalexin-resistant (100%, *n* = 10), and five strains were resistant to chloramphenicol (50%). The most frequent serotype was O113:H21 (40%), followed by O76:H19 (40%), O91:H14 (10%), and O130:H11 (10%). The *stx*1 type was detected in all isolated strains, while *stx*2 was only detected in two strains. The Stx subtype most frequently detected was *stx*1c (80%), followed by *stx*1a (20%), *stx*2b (10%), and *stx*2d (10%). All strains harbored chromosomal *bla*_AmpC_. Principal component analysis shows that BPS size, number of cattle, pet and horse, and elevation act as driver of antimicrobial usage. Logistic multivariable regression shows that recognition of diseases in animals (*p* = 0.038; OR = 9.382; 95% CI: 1.138–77.345), neighboring poultry and/or swine BPS (*p* = 0.006; OR = 10.564; 95% CI: 1.996–55.894), visit of Veterinary Officials (*p* = 0.010; OR = 76.178; 95% CI: 2.860–2029.315) and close contact between animal species in the BPS (*p* = 0.021; OR = 9.030; 95% CI: 1.385–58.888) increase significantly the risk of antimicrobial use in BPS. This is the first evidence of STEC strains circulating in BPS in Chile, exhibiting phenotypic AMR, representing a threat for animal and public health. Additionally, we identified factors acting as drivers for antimicrobial usage in BPS, highlighting the importance of integration of these populations into surveillance and education programs to tackle the potential development of antimicrobial resistance and therefore the risk for ecosystemic health.

## Introduction

Shiga toxin-producing *Escherichia coli* is considered one of the most common causes of foodborne disease worldwide, causing diarrhea with or without blood and potentially hemolytic uremic syndrome (HUS) in people (1). In the last decade, STEC has become much more prevalent in developing countries, with variations in the age distribution, geographic region and socioeconomic factors (2), which has led to its consideration as an emerging pathogen (3). Estimates indicate over 2.8 million annual acute illnesses worldwide, and up to 4,000 annual cases of HUS associated to STEC infection (4).

STEC strains are usually detected in ground beef and ready-to-eat food or drink (5), derived from domestic animals specially raised in intensive production farms (6, 7). Also, there is evidence about STEC in backyard production systems (BPS), but the information is scarce (8). BPS are considered as one of the most common forms of animal production worldwide, with particular importance in developing countries (9). These animal husbandry systems, which involve carrying out agricultural and livestock activities in a common space, constitute a part of the family farming system corresponding to a fragment of the family income source. As such, it implies that the activities and times allocated to animals breeding are conditioned by other household activities (10).

BPS are defined as small-scale production systems, not exceeding 100 animals, which are mainly poultry and pigs (11), among other species maintained (12). Their main features are the low levels of biosecurity, technological development and veterinary assistance, resulting in a close contact between humans and these animals, leading to pathogen transmission and dissemination. This could potentially increase the risk of failures in early detection of zoonotic and non-zoonotic outbreaks (11, 13–15). Ill animals from BPS are usually sold, slaughtered, and consumed, without considering the risk of zoonotic infections, increasing the risk of human infection (16). In this context, some of the most important diarrheagenic bacteria have been described in BPS throughout the world, including *Campylobacter* spp., *Salmonella enterica*, and STEC, all of which have also been associated with outbreaks in people (11, 17–21). Therefore, BPS could be an important source of pathogens to people. In this context, reports from BPS estimate STEC prevalence between 0.2 and 74% in dairy cattle (22), over 70% in sheep and goats (23), and even a 4% in captive wild birds (24), with several other reports in different animal species with close in-contact with humans (25, 26). Information about positivity to STEC in Latin America is scarce, but a report described STEC isolation in alpacas, raised under small farmer condition in Peru (25).

The identification and characterization of STEC is based in the detection of the Shiga toxins (Stx), with two types (Stx1 and Stx2), further classified into four subtypes for Stx1 (Stx1a, Stx1c, Stx1d, and Stx1e) and 12 for Stx2 (Stx2a-l) (27, 28). Nevertheless, little information is available for STEC characterization in the BPS context in Latin America, while available information worldwide reports a variable carriage of Stx virulence genes in isolates from backyard animals (8).

The extended use of antimicrobial drugs in the food animals' industries, including fish, cattle, swine and chicken, has led to an increase of the antimicrobial resistance (AMR) in zoonotic bacteria. These phenotypes may be transferred, as well as their resistance encoding genes, to humans directly by contact or throughout the food chain (29, 30). AMR in bacteria from the *Enterobacteriaceae* family is a sign of the emergence of resistant bacterial strains in the environment (31, 32), including *E. coli, Klebsiella* spp., *Proteus* spp., and *Salmonella* spp. (19, 33, 34). Additionally, significant losses in terms of morbidity and mortality have been reported due to multi-drug resistance (MDR) in bacterial infections (35, 36). Moreover, in the last 5–10 years a growing demand for “organic foods” has been reported, including animals sourced from backyard production systems (37, 38). Literature supports the importance of “organic” animal foods, particularly poultry, both in terms of food safety and its economic impact in low income populations when compared to conventional foods (10, 39). This may be due to the fact that BPS do not use antibiotics or synthetic growth promoters systematically and routinely, and its animals are fed in an open pasture system (40, 41). Additionally, the growing access to, and the use of, antimicrobials (either through prescription or non-prescription), in both people and animals, leads to an increase in multidrug resistance among several pathogens (42). Several genes have been linked to AMR in bacteria isolated from backyard animals, humans and even seafood, against tetracycline (*tet*A, *tet*B, *tet*C, and *tet*G) (43), amoxicillin, amoxicillin+clavulanic acid, ampicillin, and ceftiofur (*bla*_PSE−1_, *bla*_TEM_, and *bla*_CMY_) (44), among other resistance genes related to antimicrobials widely used (45). Thus, MDR STEC strains have been described as a major public health threat worldwide and in Chile (46, 47). In this context, Adesiji et al. (43) described an increase in the incidence of AMR in developing countries, related to inappropriate or uncontrolled use of these drugs in farming practices.

The aim of this study was to asses epidemiology of STEC strains isolated from animals raised in BPS from central Chile and AMR, in order to improve understanding and knowledge about these neglected animal population and their impact under a One Health approach.

## Materials and Methods

### Sample Collection

A total of 85 BPS were included in this study, located in Metropolitana de Santiago region during 2019. A proportional stratified random sampling approach was used, based on the six provinces included in the study area (Table 1), using a random allocation of sampling points, as previously described (11). BPS farm that breed poultry and/or pigs up to a maximum of 100 birds or 50 pigs were considered in this study.

**Table 1:** Demographic distribution of BPS and sample size by province, Metropolitana region, Chile.

**Region**	**Province**	**N^**°**^ of BPS breeding birds**	**N^**°**^ of BPS breeding pigs**	**Sample size**
Metropolitana	Melipilla	1,910	202	34
	Chacabuco	426	78	13
	Santiago	244	61	10
	Cordillera	237	29	5
	Talagante	387	36	7
	Maipo	632	92	16
Total		3,836	498	85

Poultry cloacal samples were collected using sterile swabs with Cary-Blair transport medium (Becton, Dickinson and Company, Franklin Lakes, NJ, USA). For pigs and any other animal, non-poultry, present at the BPS, rectal samples were collected under the same conditions. In a selection of BPS, based on viability, for environmental samples were collected including fresh feces, nesting material, floors of the poultry or pig, and other animal pens, using sterile swabs with Cary-Blair transport medium. All samples were labeled with the identification of the BPS and animal species, stored at 4°C and transported to the laboratory and kept refrigerated until processing.

### Sample Processing

#### STEC Isolation and Identification

Samples were processed according to protocols previously described (7). Briefly, swabs were suspended into 9 mL tryptone soy broth (Becton, Dickinson and Company, Franklin Lakes, NJ, USA), homogenized and incubated overnight at 42°C for enrichment. Subsequently, 25 μL of each culture were plated onto MacConkey agar (Becton, Dickinson and Company, Franklin Lakes, NJ, USA) plates then incubated at 37°C for 18–24 h. An aliquot from the confluent area of bacterial growth was then suspended in 500 μL of sterile nuclease-free water and boiled for 15 min at 100°C. Tubes were then centrifuged at 26,480 g for 5 min at room temperature. Concentration and quality (260/280 absorbance ratio) of the obtained extracted DNA was measured in a nanodrop (NANO-400 micro-spectrophotometer, Hangzhou Allsheng Instruments Co., Hangzhou, China). Samples with an absorbance ratio closest to the optimal range (1.8–2.0) were kept at −20°C for further analyses (48). Presence of *stx*1 and/or *stx*2 genes was assessed by PCR with primer sets and reaction conditions following protocols previously described (49). As positive control, a previously characterized STEC strain was used (STEC 97) (50), and *E. coli* ATCC 25922 as negative control. PCR products (5 μL) were separated by electrophoresis on a 2% (wt/vol) agarose gel and visualized under LED light (GelDock, Maestrogen Inc., Hsinchu City, Taiwan) by SYBR® Safe DNA Gel Stain 10,000X (Thermo-Fisher Scientific, Waltham, MA, USA). Product size was determined using Accuruler 100 bp Plus DNA ladder (Maestrogen Inc., Hsinchu City, Taiwan). For each PCR positive, a maximum of 30 colonies (*E. coli* phenotype) were individually plated onto MacConkey agar (Becton, Dickinson and Company, Franklin Lakes, NJ, USA) plates and subjected to the multiplex PCR in order to identify the colony harboring *stx* genes. If this was not possible, isolation was repeated from the confluent growing zone.

Once the colonies possessing the *stx*1 and/or *stx*2 genes were detected, they were identified as *E. coli* using the VITEK®2 system (bioMérieux) and the GN VITEK®2 card, according to the manufacturer's instructions.

### Phenotypic Antimicrobial Resistance Characterization

Minimal inhibitory concentration (MIC) analysis were performed to characterize phenotypic antimicrobial resistance using the VITEK2 system (bioMérieux, Marcy-l'Étoile, France) and the AST-GN98 card, according to the manufacturer's instructions. Clinical cut-off values were applied according to the Clinical and Laboratory Standards Institute guidelines (51). The antimicrobials (AM) used for the analyses included aminoglycosides (amikacin and gentamicin), β-lactams (amoxicillin-clavulanic acid, ampicillin, cephalexin, cefovecin, cefpodoxime, ceftazidime, ceftiofur and imipenem), folate synthesis inhibitors (trimethoprim-sulfamethoxazole), nitrofurans (nitrofurantoin), phenicols (chloramphenicol), quinolones (ciprofloxacin, enrofloxacin, and marbofloxacin), tetracyclines (doxycycline), and also cefepime, cefotaxime, ceftazidime alone, and in combination with clavulanic acid for the detection of extended-spectrum β-lactamase (ESBL). MDR was determined if an isolated strain presented resistance to three or more antibiotics of different classes (52). Intermediate strains were classified as resistant.

### Whole Genome Sequencing (WGS) and Assembly

From all isolated STEC strains, genomic DNA was extracted using the Wizard Genomic DNA purification kit (Promega), following manufacturer's instructions. Genomic DNA libraries were created using the QIAseq FX DNA library kit (QIAGEN) and MiSeq Reagent kit v3 600 cycles (Illumina), and sequencing was performed using 2 × 300-bp dual-index runs on an Illumina MiSeq at the University of Minnesota Mid-Central Research and Outreach Center. All raw FASTQ files were trimmed and quality filtered using Trimmomatic (v0.33) (53), specifying removal of Illumina Nextera adapters, a sliding window of 4 with an average Phred quality score of 20, and 36 as the minimum read length. Trimmed reads were *de novo* assembled using the Shovill pipeline (v1.0.4), which utilizes the SPAdes assembler (54), with default parameters (https://github.com/tseemann/shovill).

### *In silico* STEC Typing and Genotypic Antimicrobial Resistance

VirulenceFinder 2.0 (https://cge.cbs.dtu.dk/services/VirulenceFinder/) (55) was used to identify *stx* type and subtype genes. Molecular serotype was inferred with the SerotypeFinder 2.0 (https://cge.cbs.dtu.dk/services/SerotypeFinder/), based on the sequences of the O-antigen processing and the flagellin genes (56). Resistance genes were identified using ResFinder3.2 (https://cge.cbs.dtu.dk/services/ResFinder/) (57) and ABRicate (v.0.8.13) (https://github.com/tseemann/abricate/). These analyses were performed with a default setting of a 90% of identity threshold and 60% minimum gene length overlap, and the presence of these genes was confirmed when a coverage and identity >90% was identified.

### Epidemiological Data Collection

A survey was conducted on each BPS by a semi-structured interview with BPS owners, after they consent voluntarily to be part of this study. Data was collected in relation to infrastructure, biosecurity, animal production practices, and public health.

### Statistical Analysis

Descriptive statistics analysis was conducted to summarize data about antimicrobial use and about infrastructure, biosecurity and trade practices of BPS. BPSs were then classified as positive or negative for the presence of STEC.

To establish the influence on AM usage of animal maintenance-related variables in BPS, elevation (meters above sea level), and surface (acres), a principal component analysis (PCA) was carried out using “deftools,” “factoextra,” and “ggbiplot” packages of R statistical software (58). PCA was also performed in order to determine the existence of grouping among the same variables on BPS that report different AM management intervention (AM, medicinal herbs, mixed, no intervention). Given the nature of the dependent variable (use AM or not), PCA was used as an indicator of continuous variables to include in the multivariable logistic model.

Due to the nature of the information (binary or dichotomous outcome) a logistic regression model analysis was fitted to investigate factors that determine AM use in BPS, where the response can have only two values, representing the use (*Y* = 1) or not (*Y* = 0) of antimicrobials (AM). The construction of the model was performed following previously reported methods (11), briefly, as a first step a simple logistic regression was performed in order to select the variables to be included in further analyses, including the results from PCA. Variables with a *p* ≤ 0.15 in this preliminary analysis were selected for the multivariable logistic regression model. The model with the lowest log Likelihood Ratio Test (LRT) was selected for the final model (59), using a stepwise backward elimination procedure removing those variables whose regression coefficients were not significant (*p* > 0.05). The convergence of the models was set at *epsilon* (ε) = e^−16^, in order to present stricter conditions for determining statistically significant factors. Non-significant variables whose elimination induced a change of 20% in the regression coefficients of the significant variables when removed, were retained in the model to adjust for confounding factors. Biologically and epidemiologically coherent interactions were evaluated (60). Goodness-of-fit was assessed using the Hosmer and Lemeshow Test (61, 62). This considering the role of the misuse or misinformed use of AM in the potential generation of AMR among the circulating pathogens in these neglected animal population.

## Results

Seven hundred and twelve (712) samples were collected from animals raised in BPS (63) located in the Metropolitana region, Chile. Of these, 531 (74.6%) corresponds to hens samples, followed by 55 (7.7%) duck samples, 25 (3.5%) swine samples, 20 (2.8%) goose samples, and 81 (11.4%) samples belonging to small ruminants, horses, and other domestic animals that represent <2% of the total samples each one. A total of 20 samples (2.81%) belonging to 10 BPS (11.76%) were detected positive to STEC by PCR. Positivity to STEC was detected in 9 sheep (45%), 3 dairy cattle (15%), 3 ducks (15%), 2 goats (10%), 2 hens (10%), and 1 swine (5%). No environmental samples were detected as STEC positive. From the PCR positive samples, only 13 colonies (1.83%), each from different samples, were successfully isolated and of them 10 (1.40%) proceed to MIC and WGS analysis. Samples that have a *stx* positive PCR, but no isolation was possible, the diagnosis was considered as presumptive, because other bacterial species can also carry *stx* genes.

From the original positive samples, a total of 10 STEC positive samples were analyzed by MIC. Detail of AMR is summarized in Table 2. The STEC strains were susceptible to most of the AM included in the analysis. However, all of them were resistant to cephalexin (100%, *n* = 10) and five strains were resistant to chloramphenicol (50%).

**Table 2:** MICs of selected antimicrobials against STEC strains isolated from animals raised in BPS from Metropolitana region, Chile.

**Strain**	**RA-2**	**RA-3**	**RA-4**	**RA-5**	**RA-6**	**RA-7**	**RA-8**	**RA-10**	**RA-12**	**RA-13**
**Origin**	**Sheep**	**Sheep**	**Sheep**	**Sheep**	**Sheep**	**Sheep**	**Sheep**	**Sheep**	**Sheep**	**Cow**
Antimicrobial^*^										
ESBL	–	–	–	–	–	–	–	–	–	–
AN	≤ 2	≤ 2	≤ 2	≤ 2	≤ 2	≤ 2	≤ 2	≤ 2	≤ 2	≤ 2
AMC	≤ 2	≤ 2	≤ 2	≤ 2	4	≤ 2	≤ 2	≤ 2	≤ 2	≤ 2
AM	8	8	4	4	8	≤ 2	4	8	8	4
CN	8^+^	8^+^	16^+^	16^+^	16^+^	8^+^	16^+^	8^+^	8^+^	8^+^
CFO	≤0.5	1	≤0.5	≤0.5	1	≤0.5	≤0.5	1	1	≤0.5
CPD	≤0.25	≤0.25	0.5	0.5	0.5	≤0.25	0.5	≤0.25	≤0.25	≤0.25
CAZ	≤0.12	≤0.12	≤0.12	≤0.12	0.25	≤0.12	≤0.12	≤0.12	≤0.12	≤0.12
CFT	≤1	≤1	≤1	≤1	≤1	≤1	≤1	≤1	≤1	≤1
C	8^+^	16^+^	8^+^	4	16^+^	≤2	4	4	8^+^	4
CIP	≤0.06	≤0.06	≤0.06	≤0.06	≤0.06	≤0.06	≤0.06	≤0.06	≤0.06	≤0.06
DO	1	1	1	≤0.5	1	≤0.5	1	1	1	1
ENR	≤0.12	≤0.12	≤0.12	≤0.12	≤0.12	≤0.12	≤0.12	≤0.12	≤0.12	≤0.12
GM	≤1	≤1	≤1	≤1	≤1	≤1	≤1	≤1	≤1	≤1
IPM	≤0.25	≤0.25	≤0.25	≤0.25	≤0.25	≤0.25	≤0.25	≤0.25	≤0.25	≤0.25
MRB	≤0.5	≤0.5	≤0.5	≤0.5	≤0.5	≤0.5	≤0.5	≤0.5	≤0.5	≤0.5
FT	≤16	≤16	≤16	≤16	≤16	≤16	≤16	≤16	≤16	≤16
SXT	≤20	≤20	≤20	≤20	≤20	≤20	≤20	≤20	≤20	≤20

* *AN, Amikacin; AMC, amoxicillin-clavulanic acid; AM, ampicillin; CN, cefalexin; CFO, cefovecin; CPD, cefpodoxime; CAZ, ceftazidime; CFT, ceftiofur; C, chloramphenicol; CIP, ciprofloxacin; DO, doxycycline; ENR, enrofloxacin; GM, gentamicin; IPM, imipenem; MRB, marbofloxacin; FT, nitrofurantoin; SXT, trimethoprim-sulfamethoxazole*.

+ *Antimicrobial resistance*.

From the 13 STEC strains, 10 were successfully sequenced and upload to Enterobase repository (https://enterobase.warwick.ac.uk/species/index/ecoli) (Supplementary Material 1). Whole Genome sequences has also been deposited at GenBank under the accession JAEDXK000000000 to JAEDXT000000000, BioProject PRJNA682583. Molecular serotyping detected by WGS showed the presence of non-O157 strains, predominantly O113:H21 (40%, *n* = 4), O76:H19 (40%, *n* = 4), O91:H14 (10%, *n* = 1), and O130:H11 (10%, *n* = 1) serotypes. Additionally, *stx*1 was detected in all isolated strains, *stx*2 was only detected in two strains (Table 3). The Stx subtype most frequently detected was *stx*1c (80%, *n* = 8), followed by *stx*1a (20%, *n* = 2), *stx*2b (10%, *n* = 1), and *stx*2d (10%, *n* = 1) (Table 3). Using the ABRicate tools, all strains harbored the chromosomal *bla*_AmpC_ (100%, *n* = 10). No other AMR encoding genes were detected.

**Table 3:** Serotype and *Stx* type and subtype genes detected in STEC strains isolated from animals raised in backyard production systems from Metropolitana region.

**Strain code**	***Stx* type**	***Stx* subtype**	**Serotype**
RA-2	*stx*1	*stx*1c	O113:H21
RA-3	*stx*1	*stx*1c	O113:H21
RA-4	*stx*1	*stx*1c	O76:H19
RA-5	*stx*1	*stx*1c	O76:H19
RA-6	*stx*1	*stx*1c	O76:H19
RA-7	*stx*1/*stx2*	*stx*1a/*stx2*b	O91:H14
RA-8	*stx*1	*stx*1c	O76:H19
RA-10	*stx*1	*stx*1c	O113:H21
RA-12	*stx*1	*stx*1c	O113:H21
RA-13	*stx*1/*stx2*	*stx*1a/*stx2d*	O130:H11

**Figure 1:**
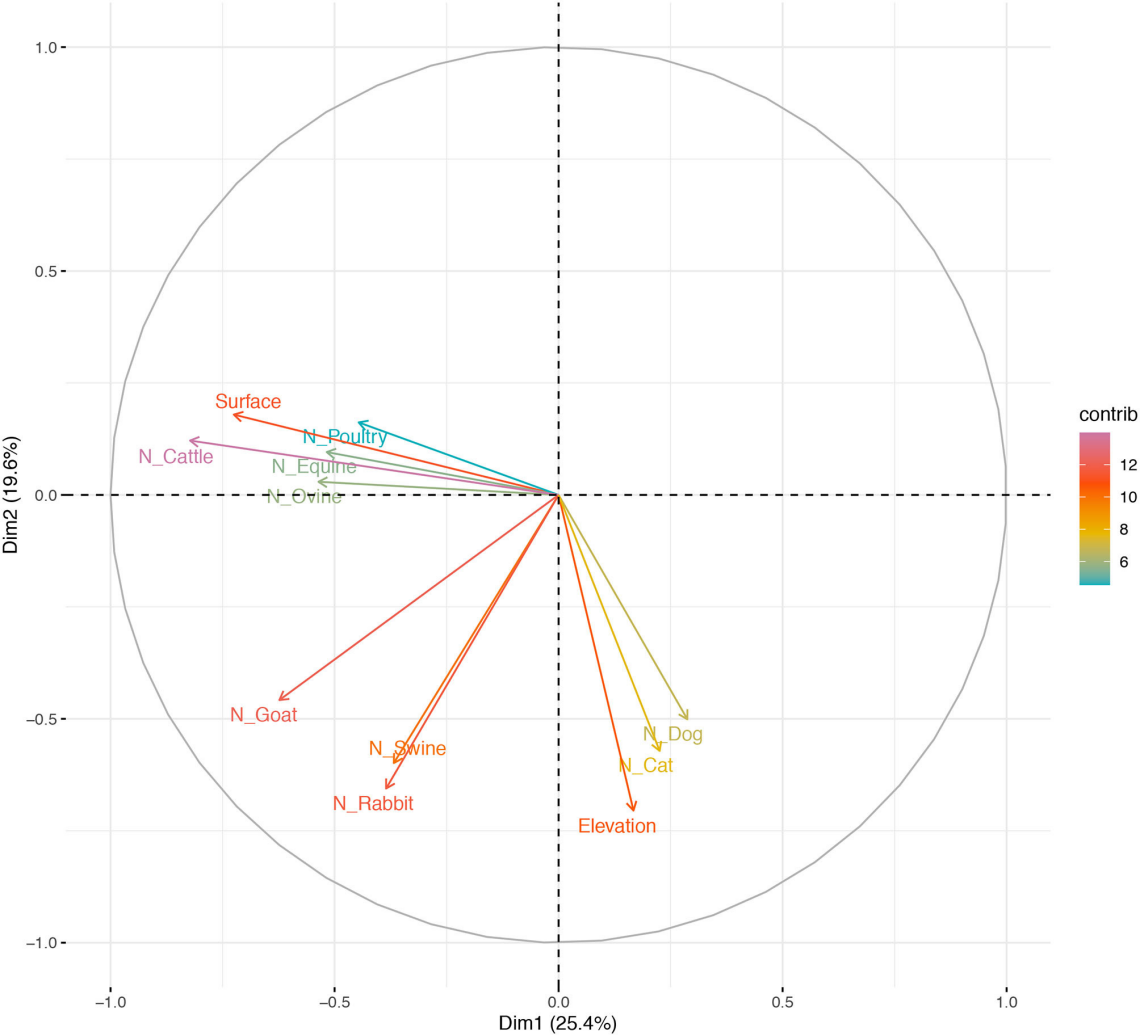
Distribution of antimicrobial use in BPS from Metropolitana region, on the first two principal components (PC) extracted from survey response on Elevation (meters above sea level), Surface (acres), N° of Poultry, N° of Cattle, N° of Equine, N° of Ovine, N° of Goat, N° of Swine, N° of Rabbit, N° of Dog and N° of Cat maintained at BPS. Space distribution of quantitative animal and BPS-related variables according to dimensions 1 and 2 (Dim 1 and 2), plotted as their eigenvectors.

**Figure 2:**
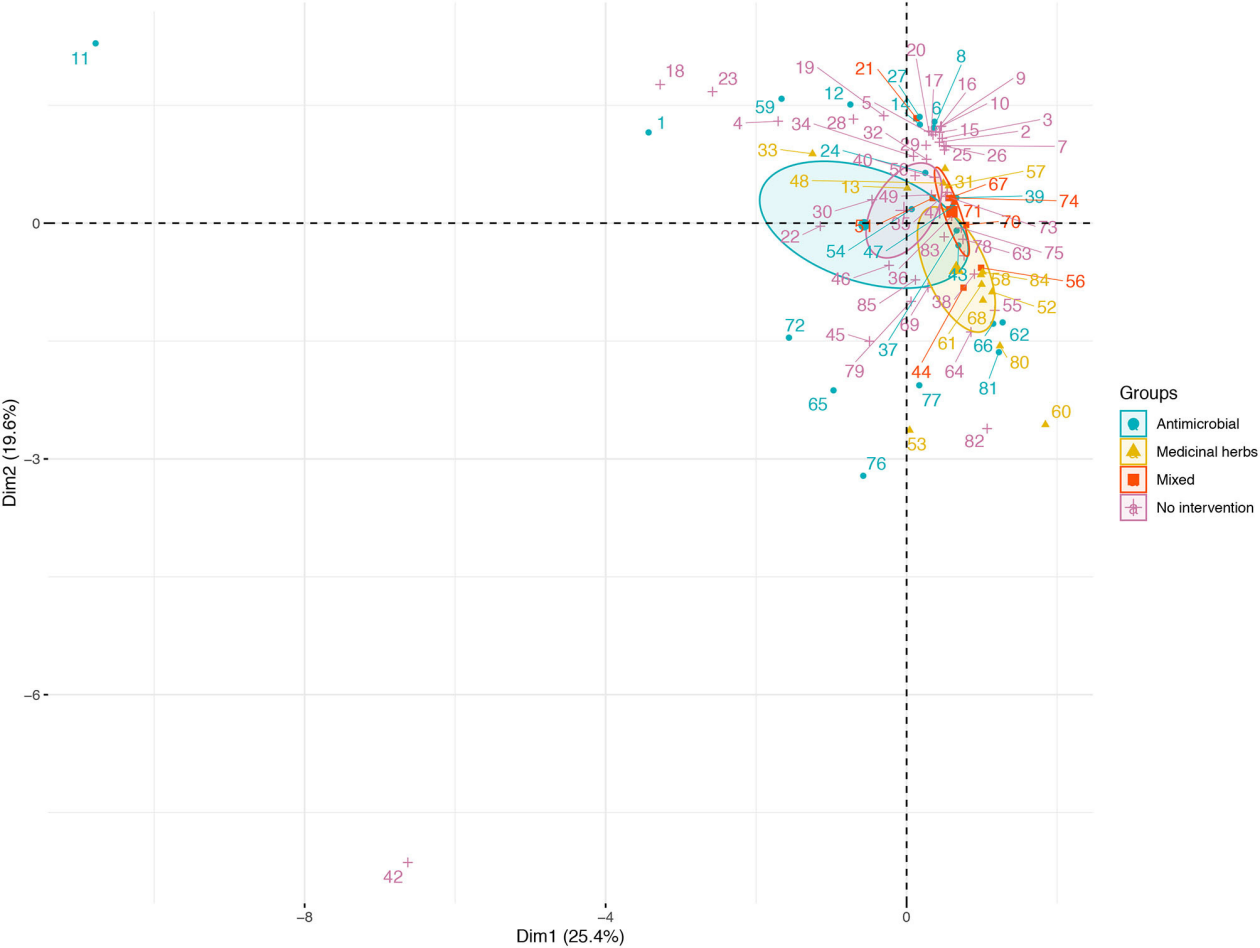
Distribution of antimicrobial use in BPS from Metropolitana region, on the first two principal components (PC) extracted from survey response on Elevation (meters above sea level), Surface (acres), N° of Poultry, N° of Cattle, N°. of Equine, N° of Ovine, N° of Goat, N° of Swine, N° of Rabbit, N° of Dog, and N° of Cat maintained at BPS. Space distribution of quantitative variables according to dimensions 1 and 2 (Dim 1 and 2), plotted as their eigenvectors and differentiated by type of intervention (AM, medicinal herbs, mixed, no intervention).

Variance (measure by eigenvalues) for the first four components (PC1–PC4), also named dimensions (dim) were >1 and they explained around 68% of the variability found in the use of AM in BPS from Metropolitana region. These components allowed us to summarize our data into multivariate linear regression analyses, without losing information or minimizing such loss. In particular, the values for these components (expressed as percentages) were 25.35, 19.60, 14.56, and 9.17%, respectively. Eigenvectors (Table 4) from these four components confirm that some of this variable are related to antimicrobial use by the BPS owner, specifically: PC1 is dominated by the N° of cattle and surface (acres), indicating that smaller BPS and the ones with lower number of cattle tend to have more chances of using AM; PC2 is dominated by the elevation of the BPS (Figure 1), this means that BPS located closer to 0 meters above sea level have more chances of using AM; PC3 is dominated the number of domestic animals (dogs and cats), indicating that the presence of pets, decrease the probability of AM usage at BPS; and PC4 is dominated by the number of horse, indicating that lower number of horses increase the risk of AM usage. No evidence of significative grouping in terms of different AM management intervention in BPS was detected (Figure 2). Furthermore, PCA results were used to determine the inclusion of quantitative variable into the multivariable model.

**Table 4:** Summary table of principal component analysis (PCA), indicating the importance of each quantitative variable of animal maintenance in BPS, elevation (meters above sea level) and surface (acres) on the use of antimicrobials, standard deviation (SD), and the percentage of explanation of variation linked to each principal component.

**Quantitative variables*^a^***	**PC1**	**PC2**	**PC3**	**PC4**	**PC5**	**PC6**	**PC7**	**PC8**	**PC9**	**PC10**	**PC11**
**Principal component eigenvectors**											
Elevation	0.17	−0.71	0.00	−0.08	0.24	−0.59	0.03	−0.18	−0.17	−0.04	0.02
Surface	−0.73	0.18	−0.43	0.22	0.03	−0.25	−0.10	0.25	−0.01	−0.11	−0.25
N° of birds	−0.45	0.16	−0.10	0.39	0.71	0.16	0.19	−0.20	0.02	0.05	0.03
N° of swine	−0.37	−0.60	0.36	−0.04	−0.04	0.02	0.50	0.33	0.12	−0.00	0.00
N° of horse	−0.52	0.10	−0.26	−0.71	−0.02	0.03	0.17	−0.30	0.11	0.07	−0.11
N° of sheep	−0.54	0.03	−0.24	0.45	−0.56	−0.14	0.17	−0.26	0.01	0.11	0.10
N° of goat	−0.62	−0.46	0.29	0.01	0.08	−0.06	−0.42	−0.01	0.35	0.07	0.07
N° of cow	−0.82	0.12	−0.21	−0.29	0.04	0.05	−0.08	0.19	−0.27	−0.07	0.24
N° of rabbit	−0.39	−0.66	0.34	0.10	−0.12	0.36	−0.13	−0.16	−0.29	−0.01	−0.15
N° of dog	0.29	−0.50	−0.70	−0.02	0.05	0.11	−0.06	0.19	−0.04	0.36	−0.01
N° of cat	0.23	−0.57	−0.65	0.05	−0.03	0.22	0.02	−0.09	0.15	−0.33	0.06
**Principal component eigenvalues**											
SD	1.67	1.47	1.27	1.00	0.95	0.81	0.75	0.71	0.60	0.53	0.42
% of Variance	25.35	19.60	14.56	9.17	8.25	5.91	5.12	4.59	3.25	2.58	1.60
Cumulative %	25.35	44.95	59.52	68.69	76.94	82.86	87.98	92.57	95.82	98.40	100.00

a *PC, Principal component; SD, Standard deviation*.

Variables retained in the final multivariable logistic regression model for antimicrobial use in BPS located in Metropolitana region can be observed in Table 5. A total of 97 variables were collected throughout the survey. Only eight variables were retained in the final model. Among them, the recognition of diseases in animals (*p* = 0.038; OR = 9.382; 95% CI: 1.138–77.345), the maintenance of poultry and/or swine in neighboring BPS (*p* = 0.006; OR = 10.564; 95% CI: 1.996–55.894), the visit of Official Veterinary Officials (*p* = 0.010; OR = 76.178; 95% CI: 2.860–2,029.315) and the close contact between different animal species in the BPS (*p* = 0.021; OR = 9.030; 95% CI: 1.385–58.888) increase significantly the risk of antimicrobial use by BPS owners. Several non-significant variables were retained in the final model, in order to account for potential confounding factors. Interaction terms were evaluated but none of them was significant at the LRT.

**Table 5:** Results of the multivariable model for antimicrobial usage in BPS from Metropolitana region, Chile.

**Variable**	**Classification**	**OR**	**95% CI**		***p*-value**
			**Lower**	**Upper**	
(Intercept)		0.006	0	0.125	0.001
Last poultry keeping	Current	Reference			
	0–1 year	0.609	0.081	4.579	0.63
	2–5 years	5.146	0.547	48.455	0.152
	> 5 years	14.345	1.279	237.054	0.063
N° of sheep		0.968	0.655	1.43	0.869
Responsible for poultry management	Family	Reference			
	Man	1.101	0.156	7.755	0.923
	Woman	0.194	0.037	0.939	0.054
Recognize	No	Reference			
diseases in animals	Yes	9.382	1.138	77.345	0.038
Veterinary/	No	Reference			
zootechnician visit	1 per year	0.113	0.004	2.971	0.191
	more than one time per year	2.673	0.112	63.824	0.544
Neighbors	No	Reference			
keep poultry and/or swine	Yes	10.564	1.996	55.894	0.006
Visit of the	No	Reference			
Official Veterinary Service	Yes	76.178	2.86	2029.315	0.01
Animal	No	Reference			
contact in BPS	Yes	9.03	1.385	58.888	0.021

## Discussion

Previous evidence reported the social and economic impact that BPS play in rural households, representing a risk for animal and human health by becoming a hot-spot of human-wildlife-domestic species contact (9, 10). One of the main features of BPS is low biosecurity measures or standards, and the maintenance of several animal species. Of these, small-scale poultry production is the most important, together with swine, cattle, and other small ruminants (11). These production systems have been linked to the occurrence of several zoonotic and non-zoonotic outbreaks worldwide (11, 14, 15, 64). BPS maintain a wide spectrum of species that harbor STEC, including cattle (22), sheep and goats (23, 65), and poultry and captive wild birds (11, 24). Positivity reported by this study (11.76%) is similar to what has been reported in productive animal species (cattle and swine) under industrialized conditions in Chile (7). As far as we know, this is the first report of STEC positivity in animals raised under BPS condition in Chile, detecting positivity in sheep (35%), cattle (25%), duck (15%), goat (10%), hens (10%), and swine (5%), highlighting the importance of BPS in terms of animal and public health. Serotypes reported by this study are commonly detected in animals or animal products (66, 67), and linked to animal and human diarrhea (68) and HUS, under particular conditions (69, 70). It is important to highlight that no O157 STEC strains were detected in this study, high morbidity, and mortality serotype linked to animal transmission (71). The *stx* subtype genes profile detected in this study, is consistent with that reported previously for most STEC isolates, both in animals from intensive farming systems and people (7, 72). Even so, it is important to highlight that O113:H21 have been linked to severe human diseases and HUS (73).

Little information is known regarding AMR of STEC strains and other enteropathogens isolated from animals raised in BPS in Latin America. Regarding phenotypic AMR in the STEC isolated strains analyzed, our results show phenotypical resistance against cephalexin in all the STEC strains isolated from animals raised in BPS, similar results to reports for cattle and swine samples under industrialized production systems in the same region of Chile (47). Even though cephalexin resistance is reported as a common feature in STEC isolates and is an antimicrobial of non-frequent use in animals or humans, non all STEC strains show this feature (74), suggesting that this resistance pattern is a threat to global health (75, 76). This could be due to the chance of exchanging resistance elements with other bacteria that share hosts with STEC or throughout other mechanisms (77). Similar resistance patterns, including β-lactamases and particularly to cephalexin, has been described for piglets, humans, free-range birds, water sources, and even STEC strains isolated from flies (78–80). Resistance to chloramphenicol was reported in five STEC strains, being different from that reported for industrialized species in Chile, where AMR was detected for a wider variety of drugs at phenotypical analysis (47). Resistance to the phenicols is mainly due to the presence of *cat* genes, encoding for chloramphenicol acetyltransferases, specific to chloramphenicol, or to the presence of *cml* genes, encoding for efflux pumps, among other mechanism, such as *nfs*B nitroreductase expression (81).

A gap in the knowledge is recognized in terms of AM usage in BPS (82, 83), leading to a potential misuse of AM in these settings. AM usage as disease preventers under BPS or similar low technification productive systems is well described (84, 85), based on the socio-economic impact of this animal housekeeping production (10). Other use reported is as growth promoters, reported in small-scale poultry production systems improving feed conversion ratios and overall productivity (63, 86), even when it has been banned in several countries, including Chile (87, 88). A lack of understanding of the public health outcomes related to BPS antimicrobial usage in this neglected population, including both animal and humans (89, 90), creates a perfect scenario for antibiotic misuse resulting in AMR generation on high impact pathogens (91). Recent reports highlights the use of AM in animal production under low and middle-income countries, a proxy to the BPS conditions, reporting that AM use was greatest in chickens, followed by swine, and dairy cattle, however, per kg of meat produced, AMU was highest in swine, followed by chickens and cattle (92), situation that could be similar under Chilean BPS conditions, if this neglected animal population was involved actively in surveillance programs of AMR or animal health (93, 94).

The PCA analysis suggest that some of the continuous variable measures during sampling, can have implications on the decision of AM usage, among them, the number of cattle raised at the BPS shows importance on the determination of AM use, as reported widely, mainly linked to the eagerness of livestock producers to meet high demand by using AM as promoters of animal growth and disease prevention, arising AMR (95), as it has been reported for *E. coli* in calves from India, observing presence of several resistance genes for carbapenems, drugs not used in food animal treatment, hence carbapenem-resistant strains in calves could possibly by originated from the natural environment or human contact (96). It has also been described to BPS pig farmers, that presents low training on animal raising, with low knowledge on AM, engaging in several irrational AM use practices (97). Surface of the BPS, measure on acres, can be a proxy of flock size or total number of animals raised in the production systems, as previously reported (98), unit size as been reported as an element of inclusion for surveillance of AM usage in animal production from low and middle-income countries (99).

Even when no significant result was detected for the maintenance of a wide diversity of animal species in a BPS and its association with AM usage, PCA and evidence suggest an important role in the maintenance and transmission of several pathogens, particularly STEC, as all these species have been reported has of STEC and *Salmonella* spp. reservoirs in Chile and worldwide (7, 11, 100, 101). In this sense logistic multivariable model highlights the role of within-BPS animal contact increasing the risk of AM usage 9.03 times, perhaps due to an increase in the probability of becoming infected with a pathogen, potentially leading to clinical signs or a decrease in the productive indicators (102), also it can be related to the presence of several potential host and therefore reservoir for a wide number of pathogens (103, 104).

Logistic multivariable regression model, also detected significant association between recognizing diseases in animals, increasing the probability of AM usage in over 9.38 times, this could be explained in a two-way direction, either BPS owner are aware of disease and also on how to treat infected animals (105, 106) or these treatments are due to a lack of knowledge on how to deal with diseases and are linked with misuse of AM, potentially leading to the development of AMR (107, 108). Linked to this risk factor, our model detected statistically significant association of AM usage with the visit of a Veterinary Officer to BPS, establishing an increase in the probability of AM usage over 70 times, it is important to highlight that Veterinary Officers only visit BPS in the presence of an outbreak of some high impact pathogens (e.g., highly pathogenic avian influenza, PRRS) (14, 15) and only return to BPS if sample results are positive to these pathogens, under this conditions, AM usage can be increased or explained due to BPS sanitary status, but should be following the guidelines and assistance of the Veterinary Officers. Model also detected significance to a 10.56 increase in the probability of AM usage when neighbors of a BPS also maintain hens or swine, the existence of animals in the vicinity plus low biosecurity measures increase the chance of pathogen transmission (11, 109) due to free animal movements, leading to the potential use of AM (110, 111), other potential explanation to this phenomena, could be related to BPS location within family groups, working under the existence of cooperation groups or by social/cultural influence of neighbors (112, 113).

This study corresponds to the first AMR report (phenotypic and genotypic) in circulating STEC strains under backyard production systems in Chile and the first epidemiological approach to understand AM usage under this animal production conditions.

## Data Availability Statement

The data generated in this study has been deposited into BioProject (accession: PRJNA682583, JAEDXK000000000, JAEDXT000000000).

## Ethics Statement

The animal study was reviewed and approved by Comité Institucional de Cuidado y Uso de Animales of the Universidad de Chile (permit code 18205-VET-UCH) for obtaining rectal samples from backyard production systems animals. Written informed consent was obtained from the owners for the participation of their animals in this study.

## Author Contributions

RA-M, NG, FS, and EP-M contributed to conception and design of the study. RA-M, NG, GA, VN, and TJ contributed with resources to the study. RA-M, EP-M, CG, BF-S, FS, BE, VF, RR, NG, JM-A, CF-F, and TJ performed the laboratory analyses. RA-M performed the statistical analysis. GA and VN revised sections of the manuscript. RA-M wrote the first draft of the manuscript. All authors contributed to manuscript revision, read, and approved the submitted version.

## Conflict of Interest

The authors declare that the research was conducted in the absence of any commercial or financial relationships that could be construed as a potential conflict of interest.
