# Supplementary material for: Antimicrobial Usage Factors and Resistance Profiles of Shiga Toxin-Producing Escherichia coli in Backyard Production Systems From Central Chile
Source: Front Vet Sci. 2021 Jan 15;7:595149. doi: 10.3389/fvets.2020.595149 (PMC7844202; doi:10.3389/fvets.2020.595149)
Supplement: Supplementary file 1 [file Table_1.DOCX]

**Supplementary Material**

**Table 1.** Strain ID and assembly barcode for Shiga toxin-producing *Escherichia coli* strain isolated from backyard production systems Quality control fulfilled the standard established by Enterobase for *E. coli*/*Shigella* (<https://enterobase.warwick.ac.uk/species/index/ecoli>).

| Name | Assembly barcode | Status | Coverage | N50 | Length | Species | Contig Number (>=200 bp) | Low Quality Bases |
| --- | --- | --- | --- | --- | --- | --- | --- | --- |
| RA-2 | ESC_MB2360AA_AS | Assembled | 36 | 103191 | 5488209 | Escherichia coli / Shigella;100.0% | 215 | 51221 |
| RA-3 | ESC_MB2361AA_AS | Assembled | 43 | 117575 | 5492211 | Escherichia coli / Shigella;100.0% | 212 | 45341 |
| RA-4 | ESC_MB2363AA_AS | Assembled | 41 | 119531 | 5502406 | Escherichia coli / Shigella;100.0% | 263 | 42146 |
| RA-5 | ESC_MB2362AA_AS | Assembled | 34 | 77314 | 5485841 | Escherichia coli / Shigella;100.0% | 262 | 53174 |
| RA-6 | ESC_MB2365AA_AS | Assembled | 28 | 56895 | 5486966 | Escherichia coli / Shigella;99.1% | 289 | 76683 |
| RA-7 | ESC_MB2366AA_AS | Assembled | 28 | 61129 | 5486234 | Escherichia coli / Shigella;99.57% | 301 | 74581 |
| RA-8 | ESC_MB2373AA_AS | Assembled | 39 | 110964 | 5446428 | Escherichia coli / Shigella;100.0% | 255 | 51109 |
| RA-10 | ESC_MB2369AA_AS | Assembled | 28 | 52582 | 5480311 | Escherichia coli / Shigella;100.0% | 276 | 77762 |
| RA-12 | ESC_MB2368AA_AS | Assembled | 31 | 88239 | 5487115 | Escherichia coli / Shigella;100.0% | 233 | 62416 |
| RA-13 | ESC_MB2367AA_AS | Assembled | 26 | 68479 | 4968858 | Escherichia coli / Shigella;99.32% | 193 | 58687 |
